# Radiologic findings of polypoid pyeloureteritis: a case report

**DOI:** 10.1016/j.radcr.2025.01.008

**Published:** 2025-01-15

**Authors:** Michael Phillipi, Cassidy Tung, Thomas Duong, Ryan O'Connell, Roozbeh Houshyar

**Affiliations:** a Department of Radiological Sciences, University of California, Irvine, Orange, CA 92868-3201, USA; b Department of Pathology and Laboratory Medicine, University of California Irvine, School of Medicine, Irvine, CA 92697, USA

**Keywords:** Polypoid, Pyelitis, Ureteritis, Pyeloureteritis, Cystitis

## Abstract

Polypoid pyeloureteritis is a rare benign exophytic mucosal lesion of the renal pelvis and ureter caused by recurrent inflammation. Risk factors include a history of radiation therapy, colovesical fistulas, or calculi. To our knowledge, we present the first documented case of polypoid pyeloureteritis with radiologic, pathologic, and clinical correlation. A 74-year-old male with a history of right papillary renal cell carcinoma status-post nephrectomy and recurrent nephrolithiasis presented for computed tomography urography for carcinoma surveillance. Computed tomography urography revealed urothelial thickening at the left renal pelvis along with small polypoid filling defects in the left renal collecting system at the ureteropelvic junction, as well as a 3mm nonobstructing stone. Biopsy of the lesion confirmed the diagnosis of polypoid pyelitis with ureteritis. Lithotripsy for stone removal was administered at the time of biopsy, and fulguration of the mass was performed. At the time of 1-year follow-up the patient denied any complaints, including urinary symptoms. Imaging was once again remarkable for extensive urothelial thickening of the left renal pelvis and proximal ureter that demonstrated subtle nodularity, consistent with known polypoid pyeloureteritis. Clinical context and pathologic findings should be considered to differentiate benign polypoid lesions from urothelial neoplasms after observing urothelial thickening and filling defects on computed tomography urography. If the lesion is non-neoplastic, fulguration combined with removal of the irritant may serve as an alternative management for surgical excision.

## Introduction

Polypoid pyeloureteritis is a rare benign exophytic mucosal lesion of the renal pelvis and ureter. Analogous lesions more commonly occur in the bladder, termed polypoid cystitis. These lesions are caused by chronic inflammation; the most common etiology for polypoid cystitis is long-standing catheterization. Other risk factors include prior radiation therapy, colovesical fistulas, or calculi. Lesions are characterized histologically by a normal or mildly hyperplastic urothelium and an inflamed, edematous stroma without metaplasia [1]. Clinical symptoms can be ambiguous, but may present with signs of bladder obstruction, hematuria, and voiding dysfunction [2]. Lesions can be identified on computed tomography (CT) and magnetic resonance imaging (MRI) from the resulting urothelial thickening and urinary tract filling defects [3,4]. Pathologic examination may help to confirm the diagnosis to differentiate this benign pathology from neoplastic urothelial tumors. However, radiological evidence of urinary tract polypoid lesions proximal to the bladder is scarce; only pathologic examinations of polypoid pyelitis have been outlined in the literature [5]. In our search of the MEDLINE/PubMed, Embase, Scopus and Google Scholar databases with the search terms “polypoid pyeloureteritis,” “polypoid pyelitis” and “ureteritis,” we did not identify any published literature detailing the radiologic findings associated with polypoid pyeloureteritis. In this case report, we aim to present the radiological findings of polypoid pyeloureteritis, while providing further clinical and pathologic correlation.

### Case report

**Fig. 1:**
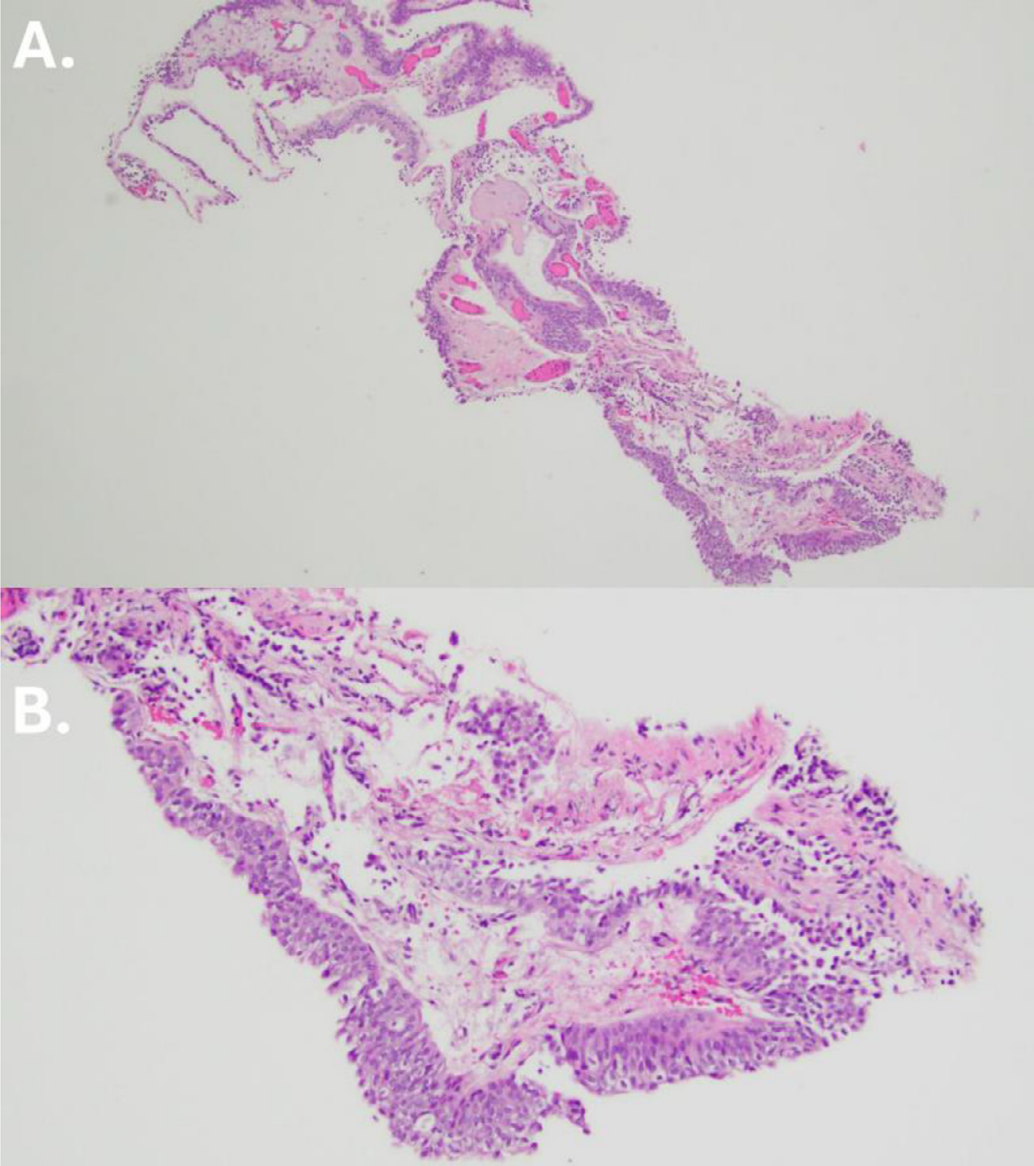
Pathology findings: Hematoxylin and Eosin stains of the ureteroscopic biopsy at 40x magnification (A) and 100x magnification (B) show polypoid protrusions without branching of papillae. The lamina propria ranges from edematous to fibrotic and contains delicate vessels with congestion. There is mild chronic inflammation. No significant urothelial atypia is present.

A 74-year-old male with a history of right papillary renal cell carcinoma (RCC) status-postnephrectomy presented to our hospital for CT urography for interval evaluation of recurrence of RCC. At the time of imaging, the patient reported feeling well and denied any symptoms, including fevers, chills, flank pain, night sweats or weight loss. The patient's other past medical conditions included a history of benign prostatic hyperplasia with bladder calculi status-post cystolithotomy and open simple prostatectomy, hypertension, and hyperlipidemia. The surveillance CT urography showed urothelial thickening at the left renal pelvis along with small polypoid filling defects in the left renal collecting system at the ureteropelvic junction, as well as a 3mm nonobstructing stone in the left upper pole. While the radiology report noted the filling defects in the renal pelvis and ureter may represent a potential primary urothelial neoplasm, pathology confirmed the benign diagnosis of polypoid pyelitis with urethritis on CT ureteroscopy with biopsy. Pathologic examination of the biopsy showed polypoid lesions and mild chronic inflammation without atypia (Fig. 1). Fulguration of the polypoid mass was performed during the procedure, followed by ureteric stent insertion. Follow-up with observation was recommended.

**Fig. 2:**
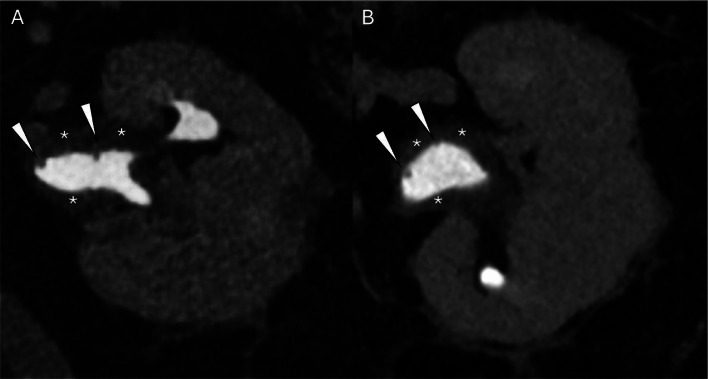
Radiology findings: Axial (A) and coronal (B) slices of CT urography in excretory phase at the level of the left kidney demonstrate extensive urothelial thickening (*) of the left renal pelvis and proximal ureter (not shown) with subtle nodular filling defects (arrowheads).

At 1-year follow-up CT urography, the patient continued to deny any symptoms. The exam was remarkable for a small left renal nonobstructive calculus without hydronephrosis. The collecting system showed stable extensive urothelial thickening of the left renal pelvis and proximal ureter that demonstrated subtle nodularity, consistent with the previous diagnosis of polypoid pyelitis with urethritis (Fig. 2). The patient showed no decline in renal function; creatinine remained stable at ∼1.25mg/dl, and estimated glomerular filtration rate was stable at ∼60mL/min/1.73m ^2^.

Given the stable findings, the patient was requested to return in 1 year for continued surveillance imaging and laboratory studies. No further management was performed.

## Discussion

Although polypoid cystitis has been well-documented in the literature, polypoid lesions more proximal in the urinary tract have been seldom reported, and no radiologic findings have been documented prior to this case report. We present the CT urography findings of a case of polypoid pyeloureteritis that shows extensive urothelial thickening of the left renal pelvis with subtle nodular filling defects. However, similar imaging findings can be identified secondary to primary neoplasms, presenting a diagnostic challenge. In fact, our case's initial radiology report suggested the filling defects may represent a primary urothelial neoplasm, advising further pathologic review of the lesion. Pathologists have also been shown to have difficulty with the accurate diagnosis of polypoid urinary tract lesions. In 1 study, pathologists misdiagnosed 41 of 155 cases of polypoid cystitis as papillary urothelial neoplasms. This study recommended the use of low magnification in pathologic examination to detect the inflammation associated with non-neoplastic polypoid lesions [2].

Clinical context can also be employed to distinguish between these two diagnoses. Known risk factors for polypoid lesions include inflammatory insults to the urinary tract, such as calculi [2]. Our patient's past medical history included recurrent uric acid and calcium oxalate nephrolithiasis, with one calculus identified on both the initial and follow-up CT urography. It is possible this case of polypoid pyeloureteritis resulted from urinary tract inflammation and obstruction secondary to renal calculi. This is supported by the findings of chronic inflammation on pathology staining. In patients with urothelial thickening of the renal pelvis and ureter, medical history of urinary tract irritation should be considered to differentiate between neoplastic and non-neoplastic lesions.

An accurate diagnosis is important for directing management of urinary tract lesions. The treatment for papillary papillomas, neoplasms of low malignant potential, and carcinomas is surgical resection via transurethral approach [6]. Although the management of non-neoplastic polypoid lesions is not well documented, literature suggests lesions may resolve after the removal of the inflammatory insult, without the need for surgical excision [7]. In this case, the patient was treated only for stone removal with lithotripsy, while the polypoid lesion was fulgurated. The patient remained asymptomatic, and the lesion remained stable on 1-year follow-up CT, providing evidence of a case of successful management without surgical excision.

## Conclusion

We present the radiologic and pathologic findings of polypoid pyeloureteritis. A review of medical history for urinary tract irritants, in addition to close examination of pathologic findings for inflammation may help differentiate benign polypoid lesions from urothelial neoplasms. If non-neoplastic, fulguration of the lesion combined with removal of the irritant may serve as an alternative management for surgical excision.

## Patient consent

Written informed consent for the publication of this case report was obtained from the patient.
